# Associations of trajectories in body roundness index with incident cardiovascular disease: a prospective cohort study in rural China

**DOI:** 10.3389/fnut.2024.1291093

**Published:** 2024-02-21

**Authors:** Shiru Zhang, Su Han, Liqiang Zheng, Yingxian Sun, Zhaoqing Sun

**Affiliations:** ^1^Department of Cardiology, Shengjing Hospital of China Medical University, Shenyang, China; ^2^School of Public Health, Shanghai Jiao Tong University School of Medicine, Shanghai, China; ^3^Department of Cardiology, the First Affiliated Hospital of China Medical University, Shenyang, China

**Keywords:** body roundness index, cardiovascular disease, trajectory, prospective cohort study, rural China

## Abstract

**Aims:**

The body roundness index (BRI) has good predictive ability for both body fat and visceral adipose tissue. Longitudinal BRI trajectories can reveal the potential dynamic patterns of change over time. This prospective study assessed potential associations between BRI trajectories and incident cardiovascular disease (CVD) in rural regions of Northeast China.

**Methods:**

In total, 13,209 participants (mean age: 49.0 ± 10.3 years, 6,856 [51.9%] male) were enrolled with three repeated times of BRI measurements at baseline (2004–2006), 2008, and 2010, and followed up until 2017 in this prospective study. Using latent mixture model, the BRI trajectories were determined based on the data from baseline, 2008 and 2010. Composite CVD events (myocardial infarction, stroke, and CVD death combined) was the primary endpoint. Cox proportional-hazards models were used to analyze the longitudinal associations between BRI trajectories and incident CVD.

**Results:**

Three distinct BRI trajectories were identified: high-stable (*n* = 538), moderate-stable (*n* = 1,542), and low-stable (*n* = 11,129). In total, 1,382 CVD events were recorded during follow-up. After adjustment for confounders, the moderate-stable and high-stable BRI groups had a higher CVD risk than did the low-stable BRI group, and the HR (95%CI) were 1.346 (1.154, 1.571) and 1.751 (1.398, 2.194), respectively. Similar associations were observed between the trajectories of BRI and the risk of stroke and CVD death. The high-stable group was also significantly and independently associated with CVD, myocardial infarction, stroke, and CVD death in participants aged <50 years.

**Conclusion:**

BRI trajectory was positively associated with incident CVD, providing a novel possibility for the primary prevention of CVD in rural regions of China.

## Introduction

Cardiovascular disease (CVD) is a major public health problem and the main cause of non-communicable disease morbidity and mortality globally, placing an increasing economic burden on residents and society (1, 2). The rising prevalence of CVD is largely due to the extended life expectancy and unhealthy lifestyles, including smoking, unhealthful dietary habits, lack of exercise participation, and obesity. Obesity is associated with the development of CVD (3). Body mass index (BMI) has been recognized as a longstanding and acknowledged measure of adiposity in clinical and epidemiological studies (4). Despite its ease of application, BMI is a rough measurement parameter that cannot distinguish the distribution of adipose tissue. Abdominal obesity defined by waist circumference (WC), is a strong predictor for the risk of CVD (5). Another problem with BMI is that some studies have found a phenomenon called the “obesity paradox,” which states that obesity defined by BMI plays a protective role in heart failure and mortality reduction (6). As a common anthropometric measure of central obesity, WC is limited by not accounting for the height and body mass of the subjects (7, 8). Therefore, we still need an effective and easily measurable indicator of obesity to identify CVD risk.

In 2013, body roundness index (BRI) was proposed as a new shape measure by Thomas et al. (9). It is based on height and WC to predict fat distribution with good ability (*R*^2^ for male body fat percentage is 0.78; *R*^2^ for male visceral adipose tissue percentage is 0.56) (9). However, the calculation method is relatively complex. Some studies showed that BRI had a superior predictive capacity on metabolic syndrome (10), and also might be used as an adipose indicator to determine the presence of diabetes and hypertension (11, 12). Zhou et al. observed a U-shaped relationship between BRI and all-cause mortality as well as cardiovascular mortality (13). At present, most longitudinal studies measure indicators at only one point in time, overlooking the potential effects of their dynamic changes over time on CVD. The group-based trajectory model can be used to analyze the dynamic process and potential dynamic change pattern of research factors over time (14), especially for anthropometric measures. Recent studies have explored the association of BMI or WC with hypertension, carotid stiffness and CVD based on group-based trajectory model (15–17). Kailuan study (18) found that BRI trajectories were significantly associated with CVD risk in urban community-based populations. Our study was based on the rural population of Fuxin County in China, which is generally characterized by heavy physical activity, a high sodium intake, and a low educational background. Fuxin County is a multi-ethnic area and participants in this region had different lifestyles and food habits from those of the participants in the Kailuan study. Currently, there are no studies focused on BRI trajectories and incident CVD in Chinese rural population.

In this longitudinal prospective study, we aimed to explore the longitudinal trajectories of BRI dynamics and assess the potential associations between BRI trajectories and the risk of CVD in the rural regions of Northeast China.

## Materials and methods

### Study population

**Figure 1:**
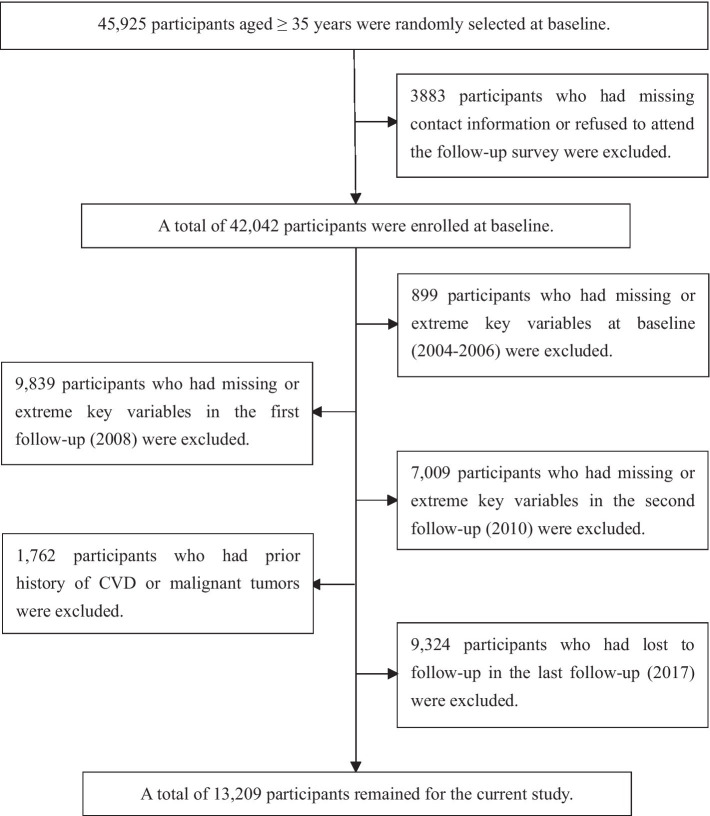
Flowchart of the study. CVD, cardiovascular disease.

This is a large-scale epidemiological prospective cohort study. From 2004 to 2006, participants aged ≥35 years were recruited with a multistage stratified cluster random sampling scheme in Fuxin County, China (including 8 towns and 84 rural villages). Eight towns were randomly selected from 5 geographic regions (northern, southern, western, eastern and central regions) based on population, and each town randomly drawn 8–12 villages from different geographical areas. A total of 45,925 participants were enrolled to collect baseline data in 2004–2006 and follow-up studies were conducted in 2008, 2010, and 2017. The detailed protocols and procedures of this study have been described previously (19, 20). Data pertaining to the participants’ anthropometrics and medical histories were assessed and recorded using standardized questionnaires and were updated during each follow-up period. In total, 3,883 participants who lost contact or refused to participate in the follow-up survey were excluded, resulting in a total of 42,042 participants at baseline. We further excluded those who had the following: (1) missing or extreme key variables at baseline, (2) missing or extreme key variables in the follow-up period (2008 and 2010), (3) a history of CVD or malignant tumors before 2010, (4) lost to follow-up in 2017. Ultimately, 13,209 participants who completed all three follow-up visits remained in the study (Figure 1).

This study was in accordance with Helsinki Declaration and approved by the Ethics Committee of China Medical University (2004PS51K). Written informed consent was obtained.

### BRI measurements

Anthropometric parameters (height, WC, and weight) were assessed and recorded using standardized procedures. Body mass was measured with calibrated platform scales (to the nearest 0.1 kg) and height with stadiometers in a standing position (to the nearest 0.1 cm), when participants wore light clothes with no shoes. After normal exhalation, use a non-elastic measuring tape to measure WC at the midpoint between the superior border of the iliac crest and the inferior costal margin on the midaxillary line (to the nearest 0.1 cm).

BRI was calculated based on a study by Thomas et al. (9):

$BRI=364.2-365.5\times \sqrt{1-\frac{{\left(\frac{wc}{2\pi }\right)}^{2}}{{\left(0.5\mathrm{height}\right)}^{2}}}$

### Data collection and measurements

All data were recorded and assessed by trained researchers. Baseline information on participants’ demographic variables, such as sex, age, education level, ethnicity, lifestyle factors (e.g., physical activity, salt intake, alcohol drinking, and smoking status), and medical history was self-reported via standardized questionnaires. In rural areas of China, salt is often used to make salty foods (as a preservative) or added to the diet as a condiment. By asking participants about the amount of salt added to their daily diet and the amount of salt-containing foods they consumed, the researchers calculated the total amount of salt consumed by a household each year, divided by the number of members in the household, to get an individual’s annual salt intake (19, 21). BMI was calculated according to body mass (kg) and height (m) (22). Blood pressure was measured in each participant by a certified and trained observer using an electric sphygmomanometer after a 5-min rest in the sitting position, and the average of the three measurements was used for statistical analysis. A baseline history of diabetes mellitus was identified through self-report, physician diagnosis, or medication prescription. A history of myocardial infarction (MI) or stroke was reported by the participants and confirmed using medical records.

### Study outcomes and follow-Up

The primary endpoint was the first occurrence of CVD, a composite endpoint that included MI, stroke, and CVD death. For incident CVD, when multiple events occurred during the follow-up period, the first event was used as the primary endpoint. Mortality information was obtained as follows (1): direct communicate with family members and (2) checking the International Classification of Diseases, Ninth Revision, Clinical Modification (ICD-9-CM) code. CVD mortality was confirmed using medical records, death certificates, autopsy reports, and information provided by family members. Details regarding the determination of MI and stroke have been described (20). Members of the endpoint assessment committee, who were blinded to the baseline risk factor information of the study participants, assessed all cardiovascular events. Participants were followed-up from the end of the trajectory evaluation period until the date of incident CVD, death, or the end of follow-up, whichever occurred first.

### Statistical analysis

Baseline categorical variables are presented as numbers (percentages) and were assessed using the χ*^2^* test. Continuous variables in the baseline are expressed as median (IQR), and were analyzed using Kruskal-Wallis test. The SAS PROC TRAJ procedure was run to group individuals with similar underlying BRI change patterns between 2004 and 2010 using a latent mixture model. This model can identify groups/categories within a population that have similar developmental trajectories and can distinguish between random and actual differences among individuals. Although each individual has a unique developmental process, the model can establish a relationship between time and latent variables through polynomial functions, allowing for the analysis of the dynamic processes of factors over time and the underlying patterns of dynamic changes. We fitted the longitudinal BRI data into the model with quadratic polynomial function parameters with groups ranging from 2 to 5, and compared the model with various functional forms of linear, quadratic, and cubic terms. The optimal fitting model was determined based on the following criteria: (a) improvement in the Bayesian information criterion; (b) proportion of membership in any single trajectory group >2%; and (c) posterior predicted probability >0.7 (23). Finally, the model with three groups was determined to be the best-fit model for BRI and was used for subsequent analysis. The longitudinal associations between BRI trajectories and risk of incident CVD were described and analyzed via Cox proportional hazards models with hazard ratios (HRs) and 95% confidence intervals (95% CIs). A proportional hazard assumption test was conducted based on the Schoenfeld residuals. Univariate and multivariate Cox regression analyses were conducted for survival analysis. A crude model was used as the univariate model. The multivariable model was adjusted for age, sex, ethnicity, education level, physical activity, current smoking status, current alcohol consumption, salt intake, and history of diabetes mellitus. A subgroup analysis was conducted to explore potential impact of age (age < 50 years and age ≥ 50 years) on the association between exposure (BRI trajectories) and risk of CVD. To check the robustness of the results, we conducted a sensitivity analysis by excluding participants who had experienced CVD in the first 2 years of the study. All statistical analyses were performed using IBM SPSS version 26.0 (SPSS Inc.) and SAS software, version 9.4 (SAS Institute Inc.). A two-sided *p*-value < 0.05 was considered statistically significant.

## Results

**Figure 2:**
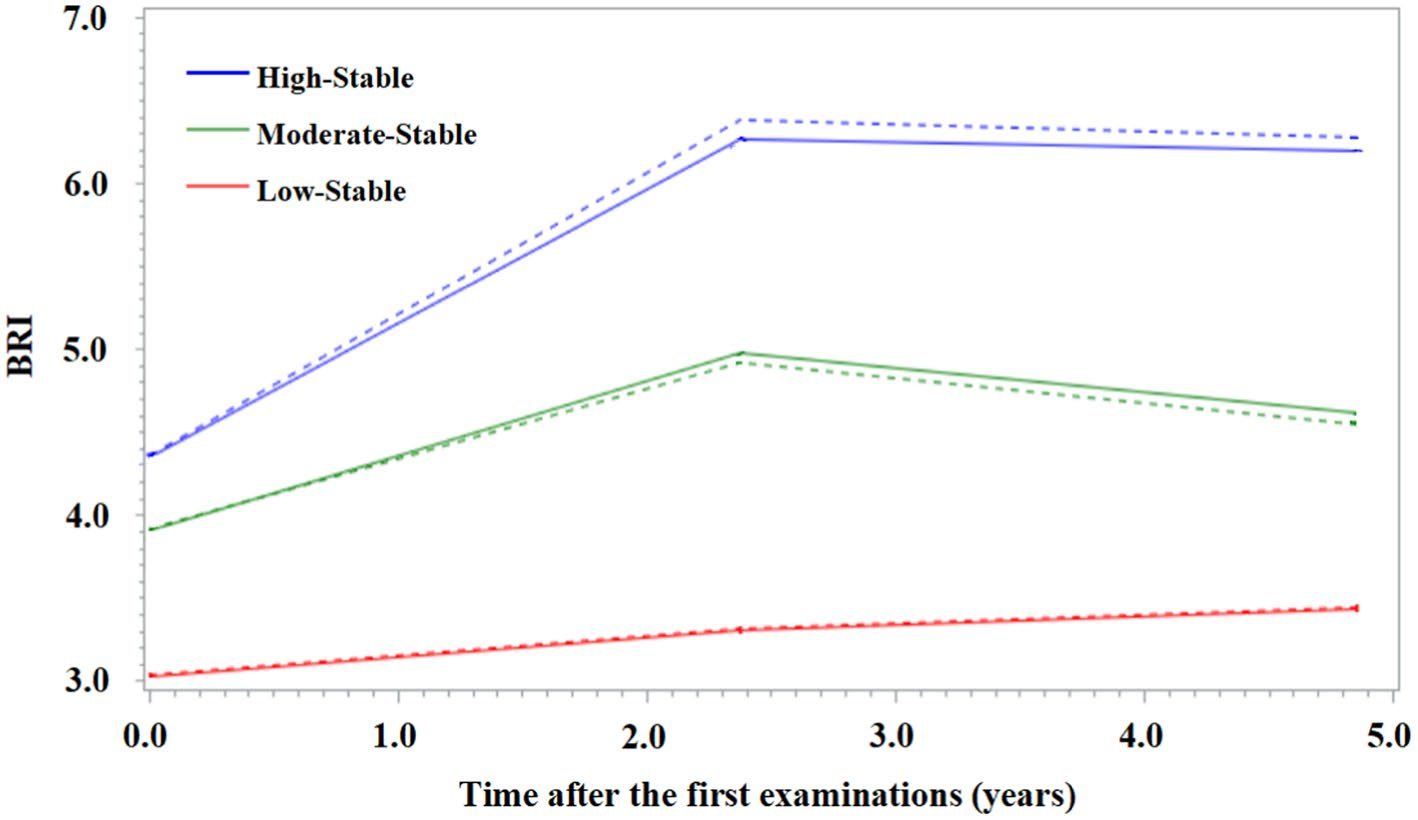
Trajectories of BRI from 2004 to 2010. BRI, body roundness index.

In total, 13,209 participants (mean age: 49.0 ± 10.3 years, 6,856 [51.9%] male) were eventually enrolled in this prospective study (Figure 1). According to the BRI measurement value and patterns of change from 2004 to 2010, the model with three BRI trajectories was determined to be the best fitting model (Supplementary Table S1), and three BRI trajectories were high-stable (*n* = 538), moderate-stable (*n* = 1,542), and low-stable (*n* = 11,129) trajectories, respectively (Figure 2). In the high-stable BRI group, BRI increased from 4.4 at baseline to 6.4 at 2008 and remained until 2010. In the moderate-stable BRI group, BRI increased from 4.1 at baseline to 5.2 at 2008 and then decreased to 4.8 at 2010. In the low-stable BRI group, BRI increased from 3.0 at baseline to 3.3 at 2008 and then slightly increased to 3.4 at 2010 (Figure 2). Detailed baseline characteristics of participants were presented in Table 1. Participants with moderate-stable and high-stable BRI trajectories were more likely to have lower physical activity, higher salt intake, higher BMI, WC, systolic blood pressure and diastolic blood pressure, were less likely to be male, have a high school or above degree or have a history of hyperlipidemia when compared with the low-stable BRI trajectory group (*p* < 0.05) (Table 1).

**Table 1:** Baseline characteristics of participants according to the trajectories of BRI.

Characteristics	BRI trajectories			*p* value
	Low-stable	Moderate-stable	High-stable	
Subjects, *n*	11,129	1,542	538	
BRI	3.0 (2.5, 3.5)	3.9 (3.2, 4.7)	4.1 (3.4, 5.0)	<0.001
Age, years	48.0 (41.0, 55.0)	48.0 (41.0, 55.0)	48.0 (41.0, 55.0)	0.913
Sex, male, *n* (%)	5,888 (52.9)	749 (48.6)	219 (40.7)	<0.001
Ethnicity, *n* (%)				<0.001
Han	8,699 (78.2)	1,066 (69.1)	420 (78.1)	
Mongolian	2,279 (20.5)	439 (28.5)	114 (21.2)	
Others	151 (1.4)	37 (2.4)	4 (0.7)	
Education level, *n* (%)				<0.001
Primary school or below	4,359 (39.2)	672 (43.6)	325 (60.4)	
Middle school	6,142 (55.2)	788 (51.1)	200 (37.2)	
High school or above	628 (5.6)	82 (5.3)	13 (2.4)	
Physical activity, *n* (%)				<0.001
Low	2,330 (20.9)	400 (25.9)	275 (51.1)	
Moderate	5,407 (48.6)	684 (44.4)	143 (26.6)	
High	3,392 (30.5)	458 (29.7)	120 (22.3)	
Current smoker, *n* (%)	861 (16.4)	110 (13.9)	39 (12.2)	0.034
Current alcohol drinker, *n* (%)	301 (5.7)	59 (7.4)	7 (2.2)	0.003
Salt intake, g/day	15.2 (11.0, 21.0)	14.0 (11.0, 19.0)	18.0 (15.0, 22.0)	<0.001
BMI, kg/m^2^	23.3 (21.9, 25.0)	25.0 (22.9, 26.7)	24.1 (22.1, 26.4)	<0.001
Waist circumferences, cm	82.0 (77.0, 87.0)	98.0 (90.0, 103.0)	106.0 (96.8, 112.0)	<0.001
Systolic blood pressure, mmHg	129.0 (120.0, 138.0)	132.0 (124.0, 140.0)	134.0 (124.0, 145.0)	<0.001
Diastolic blood pressure, mmHg	80.0 (75.0, 87.0)	82.0 (78.0, 90.0)	81.5 (76.0, 90.3)	<0.001
History of diabetes, *n* (%)	55 (0.5)	14 (0.9)	3 (0.6)	0.118
History of hyperlipidemia, *n* (%)	173 (1.6)	58 (3.8)	56 (10.4)	<0.001

Data are presented as n (%) or median (IQR).

BRI, body roundness index; BMI, body mass index.

A total of 1,382 CVD events were recorded during the follow-up period. Cox univariate analysis showed that participants in the moderate-stable and high-stable BRI trajectory groups had a higher CVD risk (HR, 1.288, 95% CI: 1.105–1.500; HR, 1.660, 95% CI: 1.329–2.074, respectively), compared with the reference (low-stable) group (Table 2). Table 2 also showed that participants in the moderate-stable and high-stable BRI trajectory groups had higher risk of stroke (HR, 1.265, 95% CI: 1.057–1.515; HR, 1.740, 95% CI: 1.348–2.247, respectively), and CVD death (HR, 1.267, 95% CI: 1.022–1.570; HR, 1.696, 95% CI: 1.251–2.301, respectively). No significant difference was observed between BRI trajectories and MI. After adjusting for age, sex, ethnicity, education level, physical activity, current smoker, current alcohol drinker, salt intake, and history of diabetes, the moderate-stable and high-stable BRI trajectory groups had higher CVD risk compared to the reference group, and HRs (95% CIs) for the other two BRI trajectory groups were 1.346 (1.154, 1.571) and 1.751 (1.398, 2.194), respectively. The adjusted HRs (95% CIs) of stroke were 1.305 (1.089, 1.565) for the moderate-stable BRI trajectory group, and 1.830 (1.413, 2.370) for the high-stable BRI trajectory group. The adjusted HRs (95% CIs) of CVD death were 1.347 (1.084, 1.675) for the moderate-stable BRI trajectory group, and 1.817 (1.334, 2.476) for the high-stable BRI trajectory group (Table 2).

**Table 2:** HRs (95% CIs) for outcomes according to the trajectories of BRI.

Cox regression hazard analysis	BRI trajectories, HR (95% CI)		
	Low-stable (*n* = 11,129)	Moderate-stable (*n* = 1,542)	High-stable (*n* = 538)
**CVD**			
Univariate	1.000 (reference)	1.288 (1.105, 1.500)	1.660 (1.329, 2.074)
Multivariable model^*^	1.000 (reference)	1.346 (1.154, 1.571)	1.751 (1.398, 2.194)
**MI**			
Univariate	1.000 (reference)	1.320 (0.964, 1.807)	1.348 (0.813, 2.234)
Multivariable model^*^	1.000 (reference)	1.439 (1.046, 1.980)	1.502 (0.901, 2.504)
**Stroke**			
Univariate	1.000 (reference)	1.265 (1.057, 1.515)	1.740 (1.348, 2.247)
Multivariable model^*^	1.000 (reference)	1.305 (1.089, 1.565)	1.830 (1.413, 2.370)
**CVD death**			
Univariate	1.000 (reference)	1.267 (1.022, 1.570)	1.696 (1.251, 2.301)
Multivariable model^*^	1.000 (reference)	1.347 (1.084, 1.675)	1.817 (1.334, 2.476)

HR, hazard ratio; CI, confidence interval; BRI, body roundness index; CVD, cardiovascular disease; MI, myocardial infarction.

^*^Adjusted for age, sex, ethnicity, education level, physical activity, current smoker, current alcohol drinker, salt intake, and history of diabetes.

**Figure 3:**
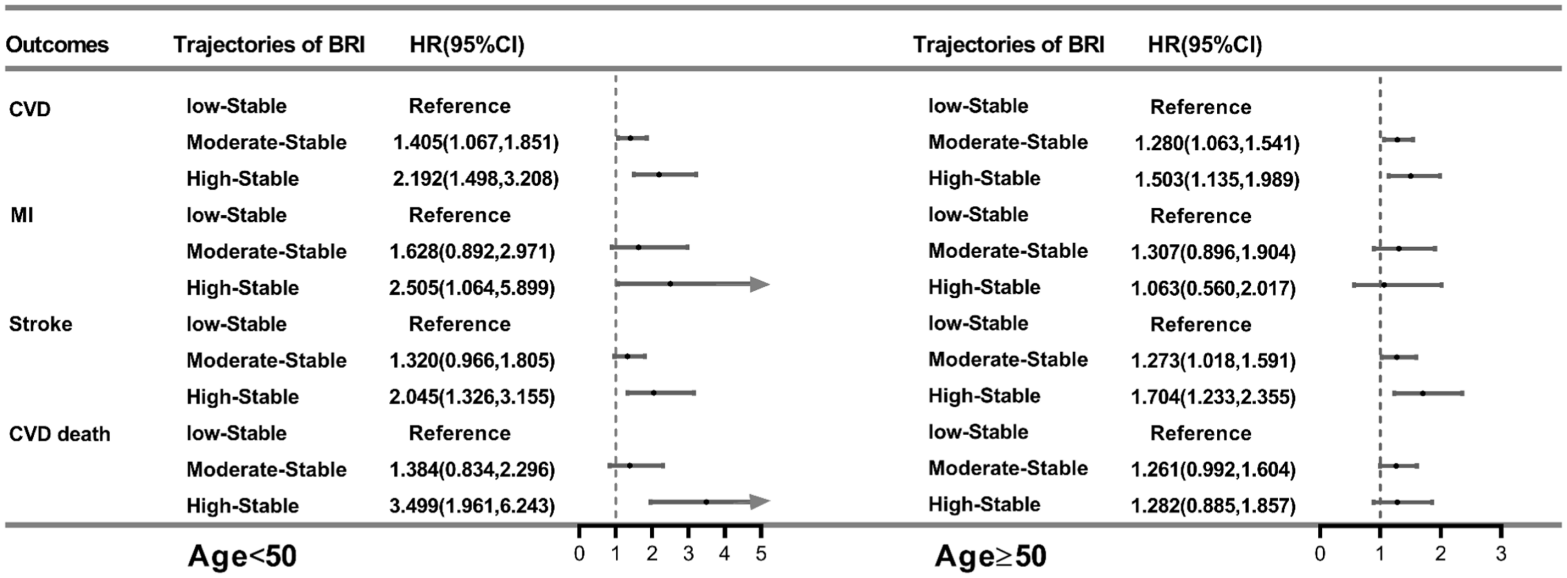
HRs (95% CIs) for outcomes per trajectory of BRI in the age subgroup. All models were adjusted for sex, ethnicity, education level, physical activity, current smoker, current alcohol drinker, salt intake, and history of diabetes. BRI, body roundness index; HR, hazard ratio; CI, confidence interval; CVD, cardiovascular disease; MI, myocardial infarction.

We also assessed the associations between BRI trajectories and the risk of endpoint events in subgroup analysis stratified by age at baseline (age < 50 years and age ≥ 50 years) in Figure 3. After adjusting for confounders, we found that compared to the group with low-stable BRI trajectory, both moderate-stable and high-stable BRI trajectory groups were significantly associated with higher CVD risk among the participants in both subgroups (aged < 50 years: HR, 1.405, 95% CI: 1.067–1.851; HR, 2.192, 95% CI: 1.498–3.208, respectively and aged ≥ 50 years: HR, 1.280, 95% CI: 1.063–1.541; HR, 1.503, 95% CI: 1.135–1.989, respectively) (Figure 3). In older adults, moderate-stable and high-stable BRI trajectory groups were just significantly independently associated with stroke, and the adjusted HRs (95% CIs) were 1.273 (1.018, 1.591) and 1.704 (1.233, 2.355), respectively. However, in younger adults, high-stable BRI trajectory group was significantly independently associated with MI (HR, 2.505, 95% CI: 1.064–5.899), stroke (HR, 2.045, 95% CI: 1.326–3.155) and CVD death (HR, 3.499, 95% CI: 1.961–6.243) in Figure 3.

The predictive performance of BRI trajectories was still strong and results had not changed substantially in sensitivity analysis after excluding participants with outcomes occurring in the first 2 years of study. The adjusted HRs (95% CIs) of CVD were 1.403 (1.187, 1.659) for the moderate-stable BRI trajectory group, and 1.728 (1.343, 2.223) for the high-stable BRI trajectory group (Table 3). Comparing to the low-stable BRI trajectory group, the other two trajectory groups was still significantly associated with stroke as well as CVD death (Table 3).

**Table 3:** HRs (95% CIs) for outcomes according to the trajectories of BRI in sensitivity analyses.

Cox regression hazard analysis	BRI trajectories, HR (95% CI)		
	Low-stable	Moderate-stable	High-stable
**CVD**			
Univariate	1.000 (reference)	1.338 (1.133, 1.579)	1.627 (1.269, 2.087)
Multivariable model^*^	1.000 (reference)	1.403 (1.187, 1.659)	1.728 (1.343, 2.223)
**MI**			
Univariate	1.000 (reference)	1.503 (1.086, 2.079)	1.419 (0.826, 2.437)
Multivariable model^*^	1.000 (reference)	1.645 (1.183, 2.289)	1.604 (0.928, 2.772)
**Stroke**			
Univariate	1.000 (reference)	1.253 (1.026, 1.531)	1.684 (1.262, 2.247)
Multivariable model^*^	1.000 (reference)	1.293 (1.057, 1.583)	1.776 (1.326, 2.379)
**CVD death**			
Univariate	1.000 (reference)	1.311 (1.035, 1.661)	1.717 (1.221, 2.416)
Multivariable model^*^	1.000 (reference)	1.411 (1.110, 1.794)	1.860 (1.316, 2.628)

HR, hazard ratio; CI, confidence interval; BRI, body roundness index; CVD, cardiovascular disease; MI, myocardial infarction.

^*^Adjusted for age, sex, ethnicity, education level, physical activity, current smoker, current alcohol drinker, salt intake, and history of diabetes.

## Discussion

In this large, rural-based study in Northeast China, we used a latent mixture model to identify three distinct BRI trajectories from baseline (2004–2006) to 2010: low-stable, moderate-stable, and high-stable trajectories. We observed that the moderate-stable and high-stable BRI trajectory groups had a higher risk of incident CVD than did the low-stable BRI group, which was independent of other potential confounders. In the subgroup analysis, BRI showed a strong association with stroke and CVD death, and MI was more pronounced in younger people. Furthermore, the above results did not substantially change in the sensitivity analysis.

The predictive performance of BRI has been established in previous studies. Rico-Martín et al. (10) illustrated that BRI was significantly associated with metabolic syndrome in different countries and ethnicities. Based on a cohort including young adults aged 21–30 years, BRI was associated with cardiovascular risk factors in rural South Africa (24). Zhang et al. found a positive correlation between BRI and dyslipidemia in women in a cross-sectional study (25), and another study found that BRI had a strong predictive effect on hypertension (26). Previous studies have used BRI from a single baseline point to assess long-term outcomes, which does not reflect the effects of dynamic process. The trajectory model (27) can repeatedly measure the studied variables, explore subgroups with different development trends, and determine the development trajectory of each subgroup; it has recently been used to study the changes in anthropometric indicators (such as BMI, WC, and BRI) over time (15–17). Wu et al. collected BRI measures on 59,278 participants from 2006 to 2012, which used to identify BRI trajectories with the latent mixture model, and they demonstrated that higher BRI trajectories were associated with the increased risk of CVD (18). We came to the same conclusion as they did. But the two studies had different definitions of CVD. In our study, CVD was defined as a composite endpoint including MI, stroke, and CVD death, while their CVD included MI, ischemic stroke, and hemorrhagic stroke. In addition, they also found that association between BRI and mortality was absent, while we confirmed that BRI was associated with CVD death in the subgroup analysis.

Although the pathophysiology of BRI in CVD requires further research, this effect may be related to known mechanisms. BRI has a better ability to predict visceral adipose tissue and body fat than traditional anthropometric indicators. Abdominal fat accumulation indicates the hypertrophy and proliferation of adipocytes, which can lead to changes in adipocyte function. Furthermore, studies have shown that BRI can significantly determine the presence of insulin resistance (IR) (10). IR can induce imbalance in glucose and lipid metabolism, which, in turn, triggers oxidative stress and induces an inflammatory response that leads to vascular endothelial cell damage (28). The results of all the above changes were structural and functional arterial wall injuries, increased arterial stiffness, impaired vasodilation, increased intima-media thickness, and increased the calcification of coronary artery, which are highly associated with future cardiovascular events (29–32). The current study also found that BRI was more effective in younger adults with MI. This result was supported by the Kailuan study, indicating that the relationship between BRI and MI is mediated by accelerating the progression of the atherosclerotic process (33).

BRI trajectory, composed of BRI values measured repeatedly at different time points, reflects the long-term patterns of individual body development. Our study showed that individuals with low-stable BRI trajectory had a lower risk of CVD and CVD death, indicating the significance of maintaining a healthy lifestyle and a lower BRI to prevent CVD in the long run. Increasing awareness of the health effects of BRI is crucial and may help self-health management to effectively reduce the incidence of diseases.

Nevertheless, this study had some limitations. First, it was conducted on rural populations in Northeast China, which limits the generalizability of the study results. Therefore, our findings must be verified in different representative populations. Second, prior history of diabetes and dyslipidemia was mainly self-reported without biochemical tests (e.g., fasting blood glucose, lipids, and glycosylated hemoglobin). Due to the poor willingness of the rural population to seek medical care, the detection rate of the diabetes and dyslipidemia may be very low. Third, although we adjusted for many important confounders, residual confounders which were unmeasured or unknown might still persist. Fourth, this study had an original population of 42,042 people; however, only 13,209 participants were recruited after exclusion because of missing data or limiting values, death, various diseases, and loss to follow-up, which may have led to different biases. Finally, our follow-up was relatively short. However, this might mean that only a few years of follow-up are needed to observe a significant association between the trajectories of BRI and CVD risk. We will continue to follow up on the study, and it is expected that there will be longer follow-up periods in the future to ensure more reliable results.

## Conclusion

In summary, BRI trajectories were strongly associated with incident CVD and CVD death, and individuals with a higher BRI trajectory had a greater CVD risk among the rural population in Northeast China. Therefore, long-term obesity plays an important role in the occurrence of CVDs. The longitudinal BRI trajectory may be a new potential indicator to provide additional evidence for the primary prevention of CVD.

## Data availability statement

The original contributions presented in the study are included in the article/Supplementary material, further inquiries can be directed to the corresponding authors.

## Ethics statement

The studies involving humans were approved by the Ethics Committee of China Medical University. The studies were conducted in accordance with the local legislation and institutional requirements. The participants provided their written informed consent to participate in this study.

## Author contributions

SZ: Formal analysis, Writing – original draft. SH: Writing – review & editing. LZ: Formal analysis, Writing – review & editing. YS: Supervision, Writing – review & editing. ZS: Conceptualization, Funding acquisition, Investigation, Supervision, Writing – review & editing.
