# Supplementary material for: Associations of trajectories in body roundness index with incident cardiovascular disease: a prospective cohort study in rural China
Source: Front Nutr. 2024 Feb 21;11:1291093. doi: 10.3389/fnut.2024.1291093 (PMC10914955; doi:10.3389/fnut.2024.1291093)
Supplement: Supplementary file 1 [file Table_1.DOCX]

Table S1. The details of the polynomial.

| Order of subgroup | | | | BIC | Average posterior probabilities | | | |
| --- | --- | --- | --- | --- | --- | --- | --- | --- |
| group 1 | group 2 | group 3 | group 4 |  | group 1 | group 2 | group 3 | group 4 |
| 1 | 1 |  |  | -60890.62 | 0.9730 | 0.8628 |  |  |
| 1 | 2 |  |  | -60605.54 | 0.9720 | 0.8600 |  |  |
| 1 | 3 |  |  | -60583.22 | 0.9701 | 0.8634 |  |  |
| 2 | 1 |  |  | -60762.15 | 0.9742 | 0.8656 |  |  |
| 2 | 2 |  |  | -60536.85 | 0.9740 | 0.8611 |  |  |
| 2 | 3 |  |  | -60515.47 | 0.9721 | 0.8639 |  |  |
| 3 | 3 |  |  | -60517.72 | 0.9721 | 0.8615 |  |  |
| 1 | 1 | 2 |  | -60319.49 | 0.9383 | 0.7314 | 0.8268 |  |
| 1 | 1 | 3 |  | -60138.83 | 0.9563 | 0.8210 | 0.7548 |  |
| 1 | 2 | 1 |  | -60327.89 | 0.9444 | 0.7648 | 0.8410 |  |
| 1 | 3 | 1 |  | -60138.83 | 0.9563 | 0.7548 | 0.8210 |  |
| 1 | 3 | 2 |  | -60146.40 | 0.9345 | 0.7672 | 0.8655 |  |
| 1 | 3 | 3 |  | -60047.73 | 0.9486 | 0.7490 | 0.8284 |  |
| 2 | 1 | 2 |  | -60216.83 | 0.9408 | 0.7427 | 0.8363 |  |
| 2 | 2 | 3 |  | -60155.01 | 0.9583 | 0.7554 | 0.8151 |  |
| 2 | 3 | 1 |  | -60111.77 | 0.9594 | 0.7478 | 0.8181 |  |
| 2 | 3 | 2 |  | -60090.13 | 0.9440 | 0.7672 | 0.8568 |  |
| 2 | 3 | 3 |  | -59988.70 | 0.9495 | 0.7478 | 0.8339 |  |
| 3 | 3 | 1 |  | -60100.40 | 0.9567 | 0.7569 | 0.8170 |  |
| 3 | 3 | 2 |  | -60068.62 | 0.9421 | 0.7600 | 0.8631 |  |
| 3 | 3 | 3 |  | -59967.19 | 0.9473 | 0.7536 | 0.8366 |  |
| 1 | 1 | 1 | 3 | -60132.60 | 0.9446 | 0.7175 | 0.7227 | 0.8352 |

Abbreviations: BIC, Bayesian information criterion.
